# Underpinning Chinese international students’ stress and anxiety during the first wave of COVID-19 outbreak: The moderating role of wisdom

**DOI:** 10.3389/fpsyg.2022.983875

**Published:** 2022-10-05

**Authors:** Alexander English, Yaxin Ding, Qionghan Zhang, Steve J. Kulich

**Affiliations:** ^1^Department of Psychology and Behavioral Sciences, Zhejiang University, Hangzhou, Zhejiang, China; ^2^Shanghai Intercultural Institute, Shanghai International Studies University, Shanghai, China; ^3^School of Business Administration, Zhejiang Gongshang University, Hangzhou, Zhejiang, China

**Keywords:** acculturation, collective coping, COVID-19 stress, anxiety, wise-reasoning

## Abstract

During the first wave of the COVID-19 outbreak, the Chinese diaspora, especially Chinese international students, were subjected to greater stress than others, because they were under pressure from both fear of infection and coping with acculturation (e.g., discrimination). Consequently, more research is needed to understand the anxiety induced by COVID-19 stresses on this specific cultural group. The main purpose of this study is to investigate the relationship between COVID-19 stress and individuals’ anxiety, and the moderating roles of Acceptance, Reframing, and Striving (ARS) coping, the family support coping strategy, and wise reasoning. To test our predictions, we collected data from 224 Chinese international students (CIS). Results indicated a strong and positive relationship between pandemic stress and anxiety. Surprisingly, both ARS and family support coping did not moderate the association between COVID-19 stress and anxiety. Instead, wise reasoning as a potential reflective coping strategy interacted with COVID-19 stress to predict anxiety. Specifically, wise reasoning predicted more anxiety when individuals perceived a low-level of COVID-19 stress, however, such a relationship disappeared when individuals perceived a high-level of COVID-19 stress. These findings about wise-reasoning extends our understanding of wisdom and how it plays a role in the context of COVID-19.

## Introduction

In January 2020, the COVID-19 pandemic broke out across China, and in the ensuing months, spread around the world. Over 601 billion individuals have been infected by the pandemic as of this writing (Sep 4, 2022), and more than 6 million have died worldwide (World Health Organization, 2021). However, looking back at that first lockdown time in early March, 2020 “the virus had spread to more than 118,000 cases and caused 4,291 deaths in 114 countries” (Bavel et al., 2020). Furthermore, many countries enacted public health policies during the first wave of the COVID-19 outbreak (e.g., lockdowns, social distancing, face-mask mandates) and due to its early ambiguity, lifestyles and personal freedoms were upended.

Against this background, the Chinese international students (CIS) faced additional grievances and stress (Shimizu, 2020; Esses and Hamilton, 2021). When COVID-19 first spread throughout China, on the one hand, CIS had to worry about family and friends back home, and on the other hand, incidents where Chinese people were violently attacked, emotionally abused, or racially blamed for the virus have been frequently reported (Li et al., 2021; Nam et al., 2021). Furthermore, CIS faced tremendous challenges due to the disruption of their spring semester in college, social isolation as a result of lockdown measures, and travel restrictions that closed borders, all of which led to uncertainty, stress, and anxiety (Xu et al., 2021). Some researchers have noted this “double blow” situation faced by CIS and found alarming increases in depression and anxiety, including traumatic experiences like switching overnight to remote learning and emotional distress from buying food or going outside (Chi et al., 2020; Lin et al., 2022). Therefore, the focus of this paper is to further understand various aspects of CIS’s psychological health at the start of the pandemic—we aim to explore the relationship between stress and anxiety and how wise-reasoning and adaptative-coping mechanisms might have played an important role in their adaptation.

## Literature review

### Stress and anxiety

Pandemic-related stress represents numerous psychosocial stressors including “severe disruptions of routines, separation from family and friends, shortages of food and medicine, wage loss, social isolation due to quarantine or other social distancing programs, and school closure” (Taylor, 2019). Bavel et al. (2020) notes that loneliness and social isolation can exacerbate stress and have harmful impacts on mental, cardiovascular, and immune health. In addition, students who are away from family members in another country also more prone to emotional difficulties (English et al., 2022).

Yet not only do the Chinese diaspora (and the CIS group among them) have to deal with pandemic stress, but also acculturative stress-stress from living in a cross-cultural environment-which can present psychosomatic symptoms such as anxiety (Berry, 2006; Szabo et al., 2017; English et al., 2021). Previous research suggests perceived discrimination is associated with heightened acculturative stress (Torres et al., 2012). Researchers have proposed that threat of an infectious illness might lead to bias and violence towards marginalized or blamed populations (Bavel et al., 2020). As the pandemic originated from China, some individuals blamed the Chinese diaspora for the appearance and spread of COVID-19 overseas (Siu and Chun, 2020). For example, in the United States, President Donald Trump labeled COVID-19 the ‘Chinese Virus’ (Forgey, 2020), prompting widespread discrimination toward Asians (Misra et al., 2020; Shimizu, 2020; Yu, 2020). Furthermore, pandemic-related discrimination perceived by the Asian group published through social media made them feel accused and distressful (Fan et al., 2021). While not all CIS may have been directly discriminated against, they could nevertheless perceive a sense of discrimination. Acculturating to new cultures can already be a stressful experience, but can be especially difficult when there is perceived discrimination (Torres et al., 2012).

Based on past research, we propose the following hypothesis:

*H1*. COVID-19 stress will be positively related to anxiety.

### Coping with stressful situations

Given the anxiety induced by the COVID-19 related stress, how to cope with this pandemic is a shared concern. In other words, effective coping strategies might reduce virus-related stress and anxiety. Coping is defined as taking proactive, inner-psychic steps to deal with demands brought on by stressful situations (Taylor and Stanton, 2007). Previous studies on coping strategies like primary (exerting control over the problem or environment) and secondary coping (adjusting oneself or cognitively reinterpreting the problem) were mainly conducted in Western contexts. Therefore, researchers have started to examine the link between coping and psychological adaptation in non-Western regions (Sahin et al., 1993; Pretorius and Diedricks, 1994; Heppner et al., 2002; English and Geeraert, 2020).

Following this goal, Heppner et al. (2006) developed the Collectivistic Coping Styles (CCS) that is “based on collectivistic Asian values and philosophies and included both primary and secondary control.” For a collectivistic culture, research has shown that Asians commonly use five coping strategies (Heppner et al., 2006): (1) Acceptance, reframing, and striving (ARS); (2) Family support; (3) Religion-spirituality; (4) Avoidance and detachment; and (5) Private emotional outlets. Some research has shown that collectivistic coping might serve as a moderator to influence the relationship between stress and psychological distress (Wei et al., 2007; Aldwin, 2009).

The current study focuses on two increasingly recognized coping strategies: (1) ARS and (2) family support.

#### ARS

Heppner et al. (2006) used the terms “acceptance, reframing, and striving” to show that people from collectivistic cultures cope with traumatic events by accepting the trauma, accommodating to reality, and changing their understanding to the tragedy. Individuals from collectivistic cultures might be more likely to adjust and reframe themselves in severe situations, trying not to trouble others around them. In consistent with such statement, Ma and Zhan (2022) found that when CIS encountered mask discrimination from Americans, they were more likely to reframe or conceal mask wearing problems (e.g., avoiding clashes) instead of challenging (or confronting) the American (lack of) mask norms. In a longitudinal cross-cultural adjustment study of Chinese international students in the United States, Wang et al. (2012) found ARS coping was most effective in reducing acculturative stress overtime. As a result, we argue that ARS coping might benefit Chinese international students who find themselves in a somewhat passive “uncontrollable state” and not capable of directly confronting pandemic related problems. Accordingly, we propose the following as our second and third hypotheses:

*H2*. The coping strategy of acceptance, reframing, and striving (ARS) will moderate the relationship between COVID-19 stress and anxiety.

#### Family support

As an integral construct or approach in collectivistic coping, family support refers to asking for help from loved ones and respected elders (Heppner et al., 2006). Collectivistic cultures believe in and practice an interdependent and inseparable relationship with family members (Markus and Kitayama, 1991), which means that looking to family members for help can be a primary choice for students to cope with stress and overcome difficulties while overseas. Kim et al. (1999) identify how Asian values like being compliant to family expectations and believing in the importance of family can help to mitigate psychological problems. In a recent qualitative study on 30 CIS in the United Kingdom, researchers found that family support functioned as a collective strategy in close collaboration between family members overcoming the increasing challenges like anxiety and fear during the early days of the pandemic (Hu et al., 2022). We argue that during pandemic times, family support will be paramount in overcoming the challenges of COVID-19 related problems, and thus propose the following additional hypotheses.

*H3*. Family support will moderate the relationship between COVID-19 stress and anxiety.

### Role of wisdom as an additional coping strategy

Wisdom, or wise-reasoning, has been characterized as a form of excellence in ethical and practical deliberation about the best course of action in complex situations (Grossmann et al., 2020; Phan et al., 2021; Zhang et al., 2022). Researchers suggest that one aspect of wise reasoning represents the sensitivity to possible changes in perspectives, recognition of limit in knowledge and comprise or willingness to accept uncertainty and different situations (Bangen et al., 2013). Wisdom is also a workable guide for people to cope with life challenges or uncertain situations (Glück, 2018). Thus, we anticipate that wisdom might be an additional coping strategy to handle the abrupt life change during a pandemic.

There is ample literature on how wise individuals make realistic decisions, recognize limits of one’s knowledge, consider multiple ways in which a situation may unfold, and recognize others’ perspectives and interests (Arriaga and Rusbult, 1998; Peetz and Grossmann, 2021). In recent years, wisdom research has shifted from a person-centric phenomenon to context dependent approach through social-ecological perspective (Grossmann et al., 2020).

From a socio-ecological perspective, wise-reasoning considers the cultural context in which individuals’ manifestation of these related qualities. One aspect of wise-reasoning is metacognitive flexibility which suggests that things are changing all the time and influenced by different factors (Grossmann et al., 2013). We argue that metacognitive flexibility will be important during an uncontrollable situation that is life changing. Perhaps, the utility of wise-reasoning might be interrelated with coping to cultivate healthy mindsets in response to traumatic events. However, a global pandemic could be by far one of the most abrupt life changes one can undergo, calling for metacognitive flexibility. Grossmann et al. (2022) interviewed over 50 world-renowned scholars in the field of behavioral and social sciences to give advice on what kind of wisdom people would need to adapt to a post-COVID era. That interview study made four recommendations toward coping with stress and uncertainty: (1) focus on long-term goals; (2) remain positive on human’s ability; (3) remain positive to intentionally master the situation; and (4) believe in social connectedness. On such an occasion, someone might be uprooted and forced to face the pandemic suddenly, therefore we argue there is a need to be realistic about and “ready to react” (e.g., be alert) quickly to the uncertainty.

Based on the above review and given that different social-ecological contexts can significantly and ultimate affect individuals, we expect that wise reasoning will play a vital role during traumatic situations in the following ways.

First, the conscientiousness engendered by wise-reasoning allows individuals to critically scrutinize ideas about what needs to be done to cope intelligently and effectively with a pandemic. This could include the importance of having personal protective equipment (e.g., masks, using hand sanitizer or disinfectant) but also adhering to strategies implemented by local health authorities and government officials.

Second, wise-reasoning may increase compliance measures such as washing hands regularly, practicing social distance, and showing concern for others, since “wise” people might actually take the pandemic more seriously and do all they can to prevent potential dangers (Peetz and Grossmann, 2021; Grossmann et al., 2022). This would include critically analyzing courses of action if one contracted COVID-19, imaginatively developing new coping strategies to stay wisely alert during the pandemic.

Simply put, wise-reasoning helps people gain insight into the emerging or changing epidemic, remain vigilant, be flexible in dealing with uncertainty, and perhaps even better embrace stress. Thus, wise reasoning, as a potential coping strategy, may weaken the relationship between stress and anxiety. Thus, we form the hypothesis below regarding the moderation effect of wisdom.

*H4*. Wise-reasoning will moderate the relationship between COVID-19 stress and anxiety.

### Significance and focus of the current study

This study sets out to understand how CIS were coping with COVID-19 while living abroad during the initial outbreak and first lockdown in 2020. We construe the pandemic as a strong and abrupt life change that is stressful and seek to explore the relationship between the stress and anxiety.

As noted in the review above, multiple coping strategies have been proposed, and some specifically for Chinese populations. Yet, there appears to be both an assumption of generalizing to collective coping and a gap of not including the wisdom orientation of Chinese. Therefore, we seek to address this gap and test whether wise-reasoning might moderate the stress-anxiety relationship in comparison to the role of other coping strategies commonly noted (ARS and family support).

To test the hypotheses proposed, the current study collected data from CIS who were scattered in Western countries like the United Kingdom and United States as well as culturally close ones like South Korea and Japan (English et al., 2021). Different cultural environments, lockdowns, and mask mandates lead to different countries’ response mechanism to pandemic control. These factors can obviously influence international students’ COVID-19 stress, coping strategies, wise-reasoning, and anxiety.

## Materials and methods

### Participants and procedure

Data was collected from 224 CIS studying abroad in 15 countries through snowballing from April 3rd to 8th, 2020, when the initial period when the COVID-19 virus was quickly spreading to and across the whole world. The researcher designed an online questionnaire on *Wenjuanxing,* a web-based survey platform, and posted it on WeChat and Weibo. To be specific, sampling occurred in three different ways. First, the researcher personally asked a group of friends who studied abroad during that period to take this survey. Second, these friends helped to forward the questionnaire to their classmates studying abroad. Third, the researcher posted the web link on moments on WeChat and Weibo, asking those who met the requirement to take the questionnaire. Some respondents had returned to China within 2 weeks of our data collection period and were also allowed to participate (requested to reflect on their fresh experiences). Outliers and bad cases were removed, including those always clicking the first option button and evidence not taking this survey seriously. Among the included sample, 145 students (64.7%) were in their host countries while 79 students (35.3%) had returned to China. Results exploring responses of the “returning home” sample are addressed in Supplemental Material S2.

Participants consisted of 142 females (63.4%) and 82 males (36.6%). The average age of the participants was 23.9 years (*SD*= 2.6; range = 18–38 years). A majority of the participants were studying for masters or equivalent degrees (141 students; 62.9%), while 55 students (24.6%) were studying for their bachelors or equivalent degrees, and 25 students (11.2%) were studying for their doctoral or equivalent degrees. Three students (1.3%) were in study abroad programs. More comprehensive details of the sample can be found in Supplemental Material S1.

Participants were studying in their host countries for an average of 1.6 years (*SD* = 2.4). The participants resided on three major continents—**Europe**: United Kingdom (34.4%) Germany (8.9%), France (4.9%), Netherlands (4.9%), Switzerland (0.4%), Spain (2.7%) Italy (1.3%); **North America**: United States (15.6%) and Canada (3.1%); **Asia Pacific**: Australia (4.5%), New Zealand (1.8%), South Korea (0.9%), Singapore (8%), Japan (8%), and Nepal (0.4%). Supplementary Figure S1 describes the sample breakdown.

### Measures

#### COVID-19 stress

The self-designed COVID-19 stress scale consisted of 10 items on a 5-point scale (1 = *Not stressful*, 5 = *Very stressful*). One sample question was “*how stressful do you feel when going out to buy daily necessities?*” The COVID-19 stress scale was designed after semi-structured interviews with 10 Chinese international students based on the real situations they encountered during the early days of the outbreak (see Supplemental Materials S3 and S4). The scale was subject to exploratory factor analysis and met thresholds (See Supplemental Material S2). Scale reliability was α = 0.84.

#### Stress-related anxiety

Students’ anxiety affecting levels of stress was assessed by the “worries” subscale from the Perceived Stress Questionnaire (Levenstein et al., 1993). The “worries” subscale asked students to what degree they agreed with each item on a 5-point scale (1 = *Not at all*, 5 = *Always*). A sample item asked of these students included “I have many worries” and “I am afraid for the future.” The scale is designed to assessed individuals’ general worries they have. The subscale was highly reliable (α = 0.88).

#### Coping strategies

ARS and family support coping strategies were assessed with the corresponding subscales of the Collectivistic Coping Styles inventory (Heppner et al., 2006), which is specifically designed within an Asian cultural orientation (details see Supplemental Material S7).

The ARS scale is the largest subscale (ARS; 11 items; a sample item: “Believed that I would grow from surviving the outbreak of the coronavirus”), which reflects a blend of acceptance, reframing, and striving. It taps into how participants interpret a stressful situation and adjust their interpretation of the stress to adapt to it. The ARS scale was highly reliable (α = 0.85).

Family Support was also assessed (FS; 6 items; a sample item: “Shared my feelings with my family”) and this subscale reflects the extent to which one seeks support from one’s family or respected elders. Items were prefaced with the following instruction:

How have you responded to difficulties of the COVID-19 outbreak? These questions below ask which strategies you are presently using and have used in the past 2 weeks.

Participants were asked to indicate their frequency on a 5-point Likert scale from 1 (*Not at all*) to 5 (*Always*). The Family Support scale was also reliable (α = 0.86).

#### Wise-reasoning

A fairly recently newly-developed situated wise-reasoning scale (SWIS) was used to examine participants’ wisdom in critically thinking about the COVID-19 pandemic (Brienza et al., 2018). After writing out their COVID-19 event response, a statement to participants requested their further response:

We would like you to continue to think about uncertainties created by the COVID-19 pandemic and report the extent to which you engaged in the following thoughts and behaviors as you have reflected on the issue.

Students responded to 10-items that were rated on a 5-point scale from 1 (Never) to 5 (Always). A sample item would be: “While I have been reflecting on this issue, I thought the issue could unfold in many different ways.” The reliability of the SWIS subscale was α = 0.78.

### Analytic strategy

The statistical analysis was performed using IBM SPSS software 23.0 (IBM Corp, Armonk, New York, United States). Before analyses were conducted, researchers first evaluated the normality of the data (Ghasemi and Zahediasl, 2012). We used Q-Q plot and the skewness and kurtosis measurement to assess the normal distribution of the data. If the data is clustered close to the line of Q-Q plot, it is normally distributed. In addition, the result of the skewness and kurtosis should be in the range of [-1.0 to 1.0] to prove the data are normally distributed.

We then start the analyses for hypotheses testing. First, a Pearson correlation and point-biserial correlation were calculated to examine the basic relationships among the variables of interest. Next, three three-step hierarchical regression analyses were performed with anxiety being the dependent variable (DV). The hierarchal linear regression was used to understand how much variance in anxiety is explained by COVID related stress and whether this is moderated by cognitive coping, familial support, or wise reasoning. Independent variables (COVID-19 stress, the two coping strategies of ARS and family support, and wisdom) were standardized. In step 1, demographic variables were added to control for gender, age, length of stay. In step 2, COVID-19 stress and moderators (two coping strategies and wise-reasoning) were entered in the model. Finally, on the third step, each interaction was introduced into the model: COVID-19 Stress × ARS Acceptance, reframing, and striving (Regression Model 1); COVID-19 Stress × Family support (Regression Model 2); COVID-19 Stress × Wise-reasoning (Regression Model 3).

## Results

### Preliminary analysis

We first check the Q-Q plot, and the data of the main variables are all clustered close to the normal-distributed line (see Supplementary Figure S3). Table 1 showed that the skewness and kurtosis of all variables were less than 1 or greater than −1, indicating that the data followed a normal distribution. Further details for other normality tests can be found in the Supplemental Material S8.

**Table 1:** Bivariate correlations of core variables.

*N =* 224	1	2	3	4	5	6	7	*M*(*SD*)	α	Skewness	Kurtosis
1.COVID-19 Stress	/							2.73(0.81)	0.85	−0.03	−0.60
2. Wise-reasoning	0.01	/						3.71(0.52)	0.78	−0.20	0.53
3. ARS Coping	−0.12	0.41^***^	/					3.84(0.67)	0.85	−0.32	0.69
4. Family Support	0.08	0.23^***^	0.35^***^	/				3.53(0.82)	0.81	−0.46	0.06
5. Anxiety	0.42^***^	0.17^**^	−0.12	0.01	/			3.17(0.98)	0.88	−0.11	−0.81
6. Age	−0.01	0.18^**^	0.04	−0.03	−0.01	/		23.89(2.64)	/	/	/
7. Gender	−0.03	−0.05	−0.03	−0.04	0.07	−0.20^**^	/	63.4%	/	/	/
8. Time in host country	−0.01	−0.01	0.01	0.06	0.08	−0.12	−0.05	190.08 (80.44)	/	/	/

** *p <0.01,*

*** *p < 0.001.*

ARS = Acceptance, Reframing, and Striving; Gender is coded (1 = Male, 0 = Female); Time in host country is in days.

Analysis revealed that COVID-19 stress was positively associated with anxiety (*r* = 0.42, *p* < 0.001) thus supporting H1. ARS coping was positively associated with familial support (*r* = 0.35, p < 0.001), wise-reasoning (*r* = 0.41, *p* < 0.001), but not stress (*r* = −0.12, *p* = 0.06) or anxiety (*r* = −0.12, *p* = 0.08). Family support was positively associated with wise-reasoning (*r* = 0.23, *p* < 0.01), but not stress (*r* = 0.08, *p* = 0.24) or anxiety (*r* = 0.01, *p* = 0.93). Wise-reasoning was positively associated with anxiety (*r* = 0.17, *p* < 0.05), but not stress (*r* = 0.01, *p* = 0.90). Results are presented in Table 1.

### Regression analyses

To examine the relationship between anxiety and COVID-19 stress, we conducted a three-step hierarchical regression to test how ARS coping (H2) would influence the relationship between stress and anxiety (see Supplementary Table S1). In the first step, anxiety was regressed on control variables. Next, we added in main variables: ARS coping and COVID-19 stress. In the final step, the two-way interaction of COVID-19 stress by ARS was not significant (*β* = −0.05, *p* = 0.43), thus rejecting H2.

Next, we tested if family support (H3) would impact the relationship between anxiety and COVID-19 stress (see Supplementary Table S2). We conducted the same three-step hierarchical regression technique. In the first step, anxiety was regressed on control variables. Next, we added in main variables: family support and COVID-19 stress. In the final step, the two-way interaction of COVID-19 stress by coping was not significant (*β* = −0.09, *p* = 0.15). Thus, contrary to our predictions, hypothesis 3 failed to be supported.

Finally, to test if wise-reasoning (H4) would impact the relationship between anxiety and COVID-19 stress, we conducted the same three-step hierarchical regression technique (Table 2). In the first step, anxiety was regressed on control variables. Next, we added in main variables: wise-reasoning and COVID-19 stress. In the final step, the two-way interaction of COVID-19 stress by wise-reasoning was significant (*β* = −0.13, *p* < 0.05). The interaction term added 2% of unique variance in anxiety, Δ*F* (1, 217) = 4.70, *p* < 0.05. Thus, Hypothesis 4 was supported.

**Table 2:** The moderating effect of wise reasoning on the relationship between COVID-19 stress and anxiety.

	step 1		step 2		step 3	
	beta	SE	beta	SE	beta	SE
Gender	−0.08	0.14	−0.09	0.13	−0.09	0.12
Age	0.01	0.03	−0.009	0.02	−0.002	0.02
Time in host	0.08	0.001	0.09	0.001	0.09	0.001
COVID-19 Stress			0.42^***^	0.06	0.43^***^	0.06
Wise-reasoning			0.17^**^	0.06	0.15^**^	0.06
Wise-reasoning × COVID-19					−0.13^*^	0.06
**model statistics**						
*R^2^*	0.01		0.22		0.23	
*ΔR^2^*	0.01		0.20		0.02	
*F*	0.85		28.23		4.70	
*df*	(3, 220)		(2, 218)		(1, 217)	

* *p < 0.05,*

** *p < 0.01,*

*** *p < 0.001.*

Gender is coded (1 = Male, 0 = Female). Wise-reasoning and COVID-19 Stress were standardized in prior.

**Figure 1:**
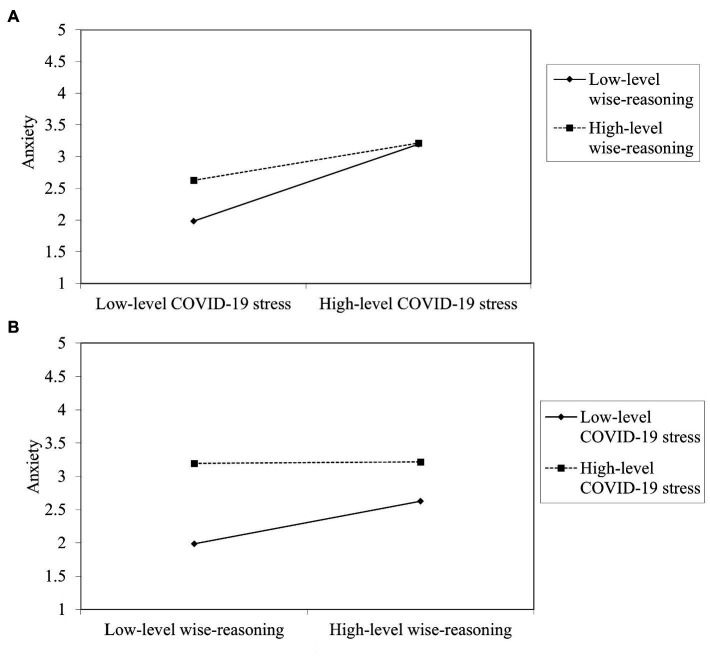
**(A)** Simple slope analysis of the moderating effect of wise-reasoning on the relationship between COVID-19 stress and anxiety; **(B)** Simple slope analysis of the moderating effect of COVID-19 stress on the relationship between wise-reasoning and anxiety.

To decompose this interaction, simple slope analyses were performed. The first simple slope analysis (Figure 1A) showed that wise-reasoning decelerated the positive association between stress and anxiety. Specifically, when participants reported low levels of wise-reasoning, stress was positively associated with anxiety (*β* = 0.61, *p* < 0.001, *d* = 1.06). However, with high levels of wise-reasoning, stress was also positively associated with anxiety, but to a smaller degree (*β* = 0.30, *p* < 0.01, *d* = 0.40). To further understand the interaction, we conducted a second simple slope analysis, swapping the independent and moderating variables (Figure 1B). Results showed that for low-level COVID-19 stress, wise-reasoning is positively associated with anxiety (*β* = 0.32, *p* < 0.001, *d* = 0.56), but in the contrast, for high-level COVID-19 stress, the relationship between wise-reasoning and anxiety is eliminated (*β* = 0.01, *p* = 0.92).

## Discussion

This study investigated how Chinese international students (CIS) responded to an abrupt lifestyle change because of the COVID-19 pandemic outbreak. We argue that these individuals experienced a “double blow” because their first exposure to the pandemic included “remote” worries and anxiety for their family members back in China, and then they later experienced first-hand lockdowns and pandemic stressors. These lockdowns led to important life changes for many people around the world. In this study, we found an unsurprising result that COVID-19 stress was associated with more anxiety especially among CIS. We further expanded COVID-19 research [as called for by Kulich et al. (2021)] by investigating the potential moderating effect of two coping strategies (ARS and family support) and an alternative strategy wise-reasoning. While we did not observe the buffering effect of ARS and family support on the relationship between COVID-19 stress and anxiety, our results found support for the wisdom-approach literature, yet in a surprising moderating way. Our major contributions are described below.

### Traditional coping did not mitigate anxiety induced by stress

Interestingly, our results show that the two coping strategies ARS and family support were not associated with anxiety. Though a surprising finding of this study, it is perhaps explainable. In a cross-national COVID-19 related study of more than 8,000 people in 67 countries (van Mulukom et al., 2020), researchers found coping mechanisms were not effective in reducing the negative effects of pandemic stress. They noted,

“…that these coping mechanisms (adaptative coping and instrumental support) are possibly not as effective as they would be in a life outside of lockdown, thus frustrating the individuals more than they contribute”(van Mulukom et al., 2020).

Our coping results follow this pattern as we find little empirical evidence in this sample that collective coping strategies like acceptance, reframing and striving (ARS) and family support aided CIS in reducing pandemic uncertainty and COVID-19 stress.

### Wise-reasoning did moderate anxiety and stress

The major contribution of this study is uncovering the moderation effect of wisdom. As stated above, wise reasoning is a tendency to reflect deeply on a social situation, that is, to consider different perspectives (Zhang et al., 2022). We discovered that CIS with a high-level of wise-reasoning appeared to be already alert to the COVID-19 situation when stress was low, thus when they perceived an increase in COVID-19 stress, it did not affect them as dramatically as CIS with a low level of wise-reasoning. For example, during that first lockdown, COVID-19 stress might have prompted people’s immediate responses of stocking up on food and personal protective equipment and dealing with the abrupt changes in schools (closure or online class) and personal life (Ben Hassen et al., 2021). Wise-reasoners tend to critically manage a tumultuous crisis like the pandemic and though they might have more anxiety at times, this can be advantageous in guiding them to good wise decisions that then help protect them from a higher level of stress.

According to earlier research, wisdom plays a crucial role in cross-cultural adaptation and dealing with upcoming obstacles in life (Bao et al., 2022) especially for those from collective traditions. Our finding is consistent and further revealed the effect of wise-reasoning and its role moderating the relationship between stress and anxiety in the context of COVID-19 pandemic. A wise-reasoner will adopt different perspectives, embrace change, or take it into careful consideration to advance the best way to better manage the drastic life shifts or stresses that COVID-19 entails. At times of low-level COVID-19 stress, these anticipatory thoughts and actions may at times cause individuals with high-level wise-reasoning to feel more anxious than individuals with low-level wise-reasoning. However, our research highlights that being vigilant is not negative and should be considered alongside other indicators, such as infection rates and response behaviors that help control the spread of COVID-19. In fact, research on Yemen health care providers offers evidence for supporting the positive link between moderate anxiety and health preventative behaviors towards COVID-19 (Alrubaiee et al., 2020). This implies that mild anxiety is positive in encouraging people to establish healthy coping actions when a crisis arises. Future research is needed to fully understand how wise-reasoning, anxiety, health preventative behaviors, and COVID-19 infection rates are related.

### Strengths and limitations

The strengths of the current study are noteworthy. First, we reported findings from a unique sample during a sensitive window of the pandemic shutdown. From the acculturation perspective, we argued that sojourners are more impacted by COVID-19 than other majority member groups. To our knowledge, very limited acculturation literature has surfaced on how sojourning groups have encountered COVID-19 life in the first wave of COVID-19 (Lee and Waters, 2021). Specifically, our study has investigated the Chinese international students and argued they were a vulnerable group as they suffered the “double blow” effect of the COVID-19 pandemic and encountered a great variety of challenges during the shutdown.

Second, we found supportive evidence of the role of wise-reasoning and its link to pandemic stress and anxiety. Specifically, when stress levels are low, wise-reasoning might promote people’s anxiety, but once stress reaches a certain point, this impact vanishes. This could be connected to wise-reasoning characteristics that extend people’s perspectives and keep them alert when faced with uncertainty. During the pandemic, an increased vigilance and recognition of the extenuating circumstances is warranted and Chinese international students or other types of sojourners should be realistic about the devasting nature of this pandemic.

However, several limitations must be mentioned.

First, while researchers collected a relatively-large sample, one third (n = 79) had already returned to China. Pandemic samples for other studies, especially those in the early stages of the pandemic, are unevenly distributed and this study is no different as we sought to collect data at that critical time from students in 15 countries. Since each country had different pandemic policies and the timing and intensity of the infection varied, responses from students naturally may differ. Different data might have been needed, other statistical tools, or a greater size of samples may have been needed to tease apart any meaningful differences.

Second, the study did not follow up the CIS’s acculturative adjustment towards the COVID-19 pandemic over time. There were many unknown, ever-changing situations that might have had an impact on students’ coping strategies, wisdom, or their psychological outcomes. Had it been possible, a longitudinal study would have been helpful to mitigate uncertainties of each population over time.

### Implications

Despite the limitations above, this study has several implications.

First, we address a noted gap by studying a sample from a large contingent of international students. Before the COVID-19 in 2020, a total of 993,367 Chinese students studied overseas at a tertiary level, among which 333,935 were in the United States, 143,323 in Australia and around 200,000 students in European countries(United Nations Educational, Scientific and Cultural Organization, 2020). These Chinese international students (CIS) have been overlooked, displaced, and in some situations misunderstood as a result of the pandemic, and are not only worthy of study, but serve as a sample that may provide some unique insights into the coping literature due to their being a from an acknowledged collective- and wisdom-oriented culture. Future research can further uncover more problems and possible solutions for what sojourners and migrant groups are experiencing in this continuing (and hopefully soon past post-) COVID-19 era.

Next, while the protective role of wise-reasoning for international students under the COVID-19 crisis has been brought to the fore, finding effective strategies to foster this feature is also a promising research direction. One of the primary techniques that researchers rely on to foster wise-reasoning is ego-decentering (i.e., self-distancing), which includes considering an issue or situation from the standpoint of a detached observer rather than one’s own perspective (Kross and Ayduk, 2011; Kross and Grossmann, 2012; Grossmann and Dorfman, 2019). Some evidence suggests that ego-decentering strategies that promote wise reasoning can enhance various behaviors, such as long-term environmental protection, healthy eating behaviors, and interpersonal perceptions (Leitner et al., 2017; Hou et al., 2018; Hussain et al., 2021). However, research on improving wise reasoning through self-decentralization strategies to protect individuals, especially international students, from the pressure of COVID-19 is scarce. As a result, future research could use this study as a starting point to investigate whether cultivating wise reasoning can be effective in reducing the epidemic’s maladjustment among international students, and even expand it to the broader community.

## Conclusion

In conclusion, this research pinpoints clear ways in which wise-reasoning might be a part of the reappraisal of stressful experiences and might be associated with a more “realistic pandemic mindset.” This research addressed an important gap in the wisdom literature by uncovering how wise reasoning can sometimes moderate the relationship between stress responses and anxiety. Possibly the wisest response in such an uncertain situation is to be adequately anxious (alert, vigilant) and become better prepared (for an adaptive response).

## Data availability statement

The raw data supporting the conclusions of this article will be made available by the authors, without undue reservation.

## Ethics statement

The studies involving human participants were reviewed and approved by Ethics Committee of Intercultural Institute at Shanghai International Studies University (Research Project Protocol # 2020-UNI-0211). The patients/participants provided their written informed consent to participate in this study.

## Author contributions

AE and YD were responsible for the concept and design of the study, data collection and analysis, interpretation of results, and writing and critical review of the manuscript. QZ was responsible for data analysis, interpretation of results, revision, and review of the manuscript. SK was responsible for providing overall program supervision and reviews of the manuscript. All authors contributed to the article and approved the submitted version.

## Funding

This research and the APC were funded by National Natural Science Foundation of China, grant number 72101232.

## Conflict of interest

The authors declare that the research was conducted in the absence of any commercial or financial relationships that could be construed as a potential conflict of interest.

## Publisher’s note

All claims expressed in this article are solely those of the authors and do not necessarily represent those of their affiliated organizations, or those of the publisher, the editors and the reviewers. Any product that may be evaluated in this article, or claim that may be made by its manufacturer, is not guaranteed or endorsed by the publisher.
