# Supplementary material for: Underpinning Chinese international students’ stress and anxiety during the first wave of COVID-19 outbreak: The moderating role of wisdom
Source: Front Psychol. 2022 Oct 5;13:983875. doi: 10.3389/fpsyg.2022.983875 (PMC9581177; doi:10.3389/fpsyg.2022.983875)
Supplement: Supplementary file 1 [file Data_Sheet_1.docx]

**Supplemental Materials**

In this supplemental document, we extend the analyses that tested our research hypotheses in the main text. We address other potential predictors (i.e., covariate variables we controlled for) that could explain increased anxiety in response to the COVID-19 pandemic. As mentioned in the main text, our hypotheses centered on the associations between overseas Chinese’ wise reasoning, coping, COVID-19 stress, and anxiety. We tested the idea that wise reasoning might enhance the relationship between COVID-19 stress and anxiety, whereas coping might not be a key factor. In the main analyses, participants’ age, gender, length of stay in host country, and COVID-19 events were considered as covariates to reduce possible confounding effects.

Here we present more background details of the study and extended analyses that we conducted in addition to the main analysis. We tested the specific influences of location, COVID-19 infections and how we devised the coding scheme, hence providing evidence to explain why we controlled for them in the main analyses in the current study.

## S1. Demographic Details: Participants

Data was collected from a total of 224 overseas Chinese students studying in different foreign countries. They answered the online questionnaire on *Wenjuanxing* that designed for this research study. Due to the severe situation of the COVID-19 pandemic outbreak in foreign countries, some respondents who had returned to China within two weeks before we started collecting data were also allowed to answer the questionnaire (fresh returnees, asked to answer from the vantage point of when they were recently overseas). Half of the participants, 145 students (64.7%) were in their host countries and 79 students (35.3%) had just returned to China. In subsection S4., we conduct analyses that tease apart these differences to make sure there are no confounding influences.

Participants consisted of 142 females (63.4%) and 82 males (36.6%). The mean age was 23.9 years (*SD* = 2.6; range = 18–38 years). The majority of participants were studying for masters or equivalent degrees (141 students, 62.9%). 55 students (24.6%) were studying bachelors or equivalent degrees and 25 students (11.2%) for doctoral or equivalent degrees. Three other 3 students (1.3%) were studying for other purposes like semester exchange programs.

### S1A. Study Location.

Participants were studying in their host countries for an average of 1.6 years (*SD* = 2.4) and were in specific host countries and continents like:

1. **Europe:** United Kingdom (34.4) Germany (8.9%), France (4.9%), Netherlands (4.9%), Switzerland (0.4%), Spain (2.7%) Italy (1.3%),
2. **North America:** United States (15.6%) and Canada (3.1%)
3. **Asia Pacific:** Australia (4.5%), New Zealand (1.8%), South Korea (0.9%), Singapore (8%), Japan (8%), Nepal (0.4%)

Due to the wide-ranging group of students, students’ host countries were grouped into non-Anglophone countries and Anglophone countries as a logical type-of-similar context. 73 students were studying in non-Anglophone countries such as Korea and Japan while 151 students were studying in Anglophone countries. In subsection S5., we conduct analyses that tease out these differences.

### S1B. During a Pandemic, Time Matters

Participants took part in this study during April 3 to April 8 in 2020. It is noticeable that the majority of students took the questionnaire from April 3 to April 6 in 2020, one day or several days after the United States publicly announced guidelines on mask usage (English, 2020).

### S1C. Participants Home Location and Role of Rice Farming

Students originated from 26 different provinces in China, with 2.7% from Anhui, Jiangxi and Tianjin separately, 6.7% from Beijing and Guangdong separately, 4.9% from Fujian, 0.9% from Gansu and Inner Mongolia respectively, 3.1% from Guizhou, Hebei, Shanxi and Liaoning respectively, 4.0% from Henan, 2.2% from Heilongjiang, Shaanxi and Sichuan separately, 1.8% from Hubei, 21.4% from Hunan, 6.3% from Jiangsu, 0.4% from Guangxi, Ningxia, Jilin and Chongqing respectively, 5.8% from Shandong, 6.3% from Shanghai and 5.4% from Zhejiang.

China is a large country with major cultural differences between north and south. In China, agricultural legacies – rice versus wheat – ingrain psychological differences between the two types of populations (Talhelm et. al, 2014). This difference has shown that people who grew up (not necessarily farm) in historically rice farmed regions of China demonstrate behaviors more closely aligned with collectivistic cultures (e.g., Japan), while wheat and millet farming regions tend to illicit behaviors more commonly found in individualistic cultures (e.g., America, Germany). One previous study has found people who move between rice-wheat regions might change coping styles to adapt to the rice-norm or wheat-norm (English & Geeraert, 2020). Also another more relevant study, found people in rice-areas exhibit more wisdom qualities (Wei & Wang, 2020).

We tested the idea that people from rice-farming areas might be wise-reasoners. However, the result showed that less rice-farming predicted more wisdom among CIS ([TableS13](#TableS11)). We ran a two-step hierarchical regression to examine the relationship between two variables: Prefecture rice farming and wisdom after controlling for current location, study location, education and gender. In the first step, wisdom was regressed on control variables which was not significant; *F*(218) =.74 , *p* =.599. Then, the main variable prefecture rice farming was added, which improved the model significantly, Δ*F*(217 ) = 4.19, *p* < .001. In this case, prefecture rice farming (β = -.14, *p* = .042) predicted less wise reasoning. This is beyond the focus of this study, but in the future researchers should consider the effect of regional cultures like rice (interdependent farming) vs. wheat (independent farming) and how they impacted COVID-19 psychological responses. Even if Chinese international students are studying in around the globe, historical “home” agricultural legacies still might impact how they reason during this pandemic.

### S1D. Procedures

Since we were interested in the COVID-19 experiences of overseas Chinese students, sending online questionnaires was the most effective and safe approach to collect data during the pandemic. The questionnaire was translated into Chinese and had been re-examined through back-translation. No major issues or questions about the wording emerged.

A pilot study was conducted by sending an established *Wenjuanxing* online questionnaire with research-related questions to 20 samples who were identified as overseas Chinese students, prior to issuing the real survey. The collected questionnaires were analyzed to qualify the validity and accuracy of the research by asking the following questions: (1) whether the sequence of the questionnaire’s items were delivered in an appropriate way, (2) whether the items were easy to understand without ambiguity, (3) whether the items would provide answers that were relevant to research questions and (4) whether the reliability and validity of each scale in the questionnaire could meet the objective of the current study. No issues surfaced.

The questionnaire has six sections: (a) demographic information, (b) COVID-19 issue description, (c) wise reasoning questions, (d) pandemic stress scale, (e) collectivistic coping, and (f) psychological distress scales. Snowball sampling was the major method to collect data by asking friends, teachers and social platforms for help. The survey was carried out mainly through communication via WeChat. While completing the questionnaire, participants were made clear about the purpose of the survey and were free to stop at any time. Students were informed that their answers would only be used for this research study and only fully completed surveys were used in analyses. Lastly, each participant would be rewarded with a completion award of 5 RMB (around 75 cents) for their completed a response. The survey took respondents around five minutes.

## S2. COVID-19 Stress Scale

The factor analysis, centralized tendency and the mean of eleven COVID-19 stress were first examined in the study. The purpose was to see the main stressors that overseas Chinese students endured during the outbreak of the COVID-19 pandemic and how scattering these stressors might be.

The COVID-19 stress scale was a self-designed measure based on the current pandemic-related threatening situations ([Table S4](#TableS2)). The COVID-19 stress scale was divided into two factors. COVID-19 Stress item 11 (“Have problems in finding an internship or job”) did not load in Factor 1 nor Factor 2. Therefore, COVID-19 Stress item 11was not stable and typical enough to describe Chinese international students’ real-life stress during the COVID-19 pandemic. This is because data was collected from diversified countries where the internships and job searching issues can be quite different, also our target population did not consist of all Chinese international students who were searching for jobs and internships.

COVID-19 Stress item 6 (“Have to stay indoors”) cross-loaded in Factor 1 and Factor 2. Through face validity, COVID-19 Stress item 6 is more related to Factor 2 than Factor 1. After analysis, COVID-19 Stress item 1 (“have to wear masks”) to COVID-19 Stress item 6 (“have to stay indoors”) were robustly related to Factor 2, which can be described as daily-life stress. COVID-19 Stress item 7 (“School shutdown”) to COVID-19 Stress item 10 (“Have problems in finding/staying in my accommodation”) were robustly associated to Factor 1, or school-related stress.

The result shows that COVID-19 Stress item 5 (“Read the overseas news on the spreading of the outbreak”, *M* = 3.20, *SD* = 1.14) and COVID-19 Stress item 8 (“Finish academic assignments or encounter graduation issues”, *M* = 3.05, *SD* = 1.29) imposed the greatest pressure on students, followed by COVID-19 Stress item 8 (“Unable to return to China”, *M* = 2.99, *SD* = 1.39), COVID-19 Stress item 3 (“Feeling unsafe in this city”, *M* = 2.85, *SD* = 1.29) and COVID-19 Stress item 4 (“Going out to buy daily necessities”, *M* = 2.85, *SD* = 1.21). COVID-19 Stress item 10 (“Have problems in finding/staying in my accommodation”, *M* = 1.82, *SD* = 1.12) imposed lower stress to students compared with other stressors. In general, overseas Chinese students were mostly stressed and affected by media news on the COVID-19 virus and their own study tasks.

## S3. Description on Traumatic Stories

Respondents were asked to describe specific events that they encountered related to the COVID-19 pandemic. Both their emotions and event description were coded by three bilingual research assistants. Some students expressed several layers of emotions and shared more than one traumatic or the COVID-19 related event. We conducted a separate code for these individuals.

In terms of traumatic story coding, the entire sample was valid (*N* = 224). Researchers applied the method of descriptive coding (Wolcott, 1994). This method mainly focuses on nouns to document the specific topic of an event. Based on Tesch (1990), the topic is “not abbreviations of the content, instead it is what is talked or written” (p. 119).

First, since participants’ descriptions were diverse, researchers transcribed the contents of their events and grouped them into 52 types of contents. In this case, the researchers coded the degree of trauma. In total, 154 students described *one* event (68.8%), 46 students described *two* events (20.5%), nine students described *three* events (4.0%) and four students described *four* events (1.8%).

Second, using the coded topics, researchers extracted four main themes from the 52 content codes. The result shows that 61 respondents described “school-related event” (26.3%), 66 students talked about “personal protective equipment (PPE) related event” (29.4%), 51 mentioned “buy daily necessities event” (23.2%) and 46 students mentioned “interpersonal relationship event” (21.1%). These results clearly show that students were greatly concerned about their school life and safety, interpersonal relationships or clear lack of them due to social distancing, daily health hygiene protection like buying masks, disinfectant, and sanitizers and buying daily necessities or the struggle of not being able to get the supplies needed.

An important question is to test if people with certain traumatic experiences demonstrate higher levels of wise-reasoning or are using coping to a greater degree? Also how are traumatic stories associated with psychological outcomes? We conducted between groups testing, and found no differences between the four events, (*x* ^2^(3)> = 3.171, *p* = 0.205).

### S3A. Location Matters, Especially during a Pandemic

The location change could also be meaningful and could influence students’ COVID-19 traumatic stories. Obviously, traumatic stories will differ based on if a student is living in the heart of the epicenter like in Italy in the spring compared to living in a country like Australia where infections were not as bad. Therefore, we tested this idea by and spotted the differences between students aboard and those who returned to China ([Figure S2](#FigS2)). In total, 145 students were still in their studying countries while 79 students were just relocated back to China. Another marked result of this is that 29% students who were in studying countries described more about a “school-related event” than students (15.2%) who were back to China (logically, as they were still in those school situations).

Results show that respondents reported different stories based on different current locations. Students still living abroad reported significantly more school related (33%) compared to students who had returned back in China (15%) *F*(3,220) =11.23, p = .001, *η*^2^=.049). Interpersonal conflicts, buying PPE, and buying daily necessities were not significantly different (*p*s > .30)

Generally across all group, respondents reported the most stories about their academic life or graduation plans as being badly impacted by the COVID-19 outbreak. Besides, other frequently mentioned traumatic events ranged from negative interpersonal relationships with local citizens to having difficulties buying daily necessities and health protective equipment, each in varying ways considered as among those students’ major stressors (See [Figure S2](#FigS2)).

## S4. Emotional Coding of the Traumatic Stories

A working definition of emotion is given by Goleman (1995), who defines an emotion as “a feeling and its distinctive thoughts, psychological and biological states, and range of propensities to act” (p. 289). Therefore, emotion coding is helpful to get hold of respondents’ feelings towards the outbreak of the pandemic and how their feelings lead to other relevant behaviors. Plutchik (1982) defines eight basic human emotions: fear, anger, sadness, joy, disgust, surprise, trust and anticipation. These were applied in the current emotion coding to stratify respondents’ emotional response.

Based on the inter-rater coding, 220 participants described specific emotions related the event they described, 195 students revealed *one* emotion (87.1%), 18 of them revealed *two* emotions (8.0%) and seven participants expressed *three* different emotions (3.1%). After recoding their general emotions as one variable, the result shows that 134 students reported “fear” (59.8%), 56 students had “neutral” feelings (25.0%), 16 students felt “sadness” (7.1%), 12 students felt “anger” (5.4%) and only two students had a sense of “joy” (0.9%).

It is reasonable that the majority of participants had negative feelings. They were fearful that they could not buy enough PPE to protect themselves, felt angry when foreigners discriminated against them and felt sad that their school life routines were totally disrupted. The students who had neutral emotion were not influenced much by the outbreak in their location or they just accepted the current change. However, there were still two students who had a positive feeling, “joy”. Based on their event description, they tried to accept the situation and reframe their understanding of the pandemic outbreak, which is why they could be joyful and less stressful.

Two major findings emerged from the emotional coding responses:

(1) Students’ negative emotional feedbacks on the COVID-19 event description revealed a sense of anxiety. As noted above (following Goleman, 1995, p. 289), besides event coding, students’ emotions were also transcribed to interpret their exact emotions behind the events (related to the “range of propensities to act”). The emotion coding captured respondents’ feelings towards the pandemic outbreak and how their feelings lead to relevant behaviors ([Table S5](#TableS3)). In this case, most CIS uncovered the feeling of fear, sadness and anger based on Plutchik (1982)’s classification on human emotions.

(2) Few students (only two of them) reported positive emotions like “joy” and “anticipation” while facing the COVID-19 stress. Noting they were attempting to manage COVID-19 stress using a cognitive positive reprisal as a means to understanding of the pandemic outbreak. This can possibly explain why coping strategies like ARS coping might not be helpful considering the severity of COVID-19 context (an abnormal and intensified coping situation).

## S5. Study Location: Anglophone vs. Non-Anglophone

To further unpack the potential confounding influence of the location where students were studying, we conducted *T*-tests and Pearson’s correlations. We explored the idea that there might be potential variations in relationships among the COVID-19 stress (COVID-19 daily and COVID-19 school), wisdom, coping strategies (ARS and family support), and anxiety. The subsample was divided into “Studying in an Anglophone Country” and “Studying in a non-Anglophone Country.”

First, based on the Pearson’s correlations analysis in [Table S6](#TableS4) and [Table S7](#TableS5), students who were studying in Anglophone countries showed a more positive association between COVID-19 stress and anxiety, *r*(151) = .50, *p* < .001, compared to those in non-Anglophone countries, *r*(73) = .43, *p* < .001. Also, students in Anglophone countries (*t*(224) = -2.48, p = .013, *d* = -.33; *M*_worries_ = 3.28, SD_worries_ = .91) reported more anxiety than those in non-Anglophone countries (M_worries_ = 2.94, SD_worries_ = 1.08). This difference is marked in [Table S8](#TableS6), while other variables showed no significant results.

Second, according to [Table S6](#TableS4), statistics showed that non-Anglophone students showed positive correlation between “family support” and wisdom *r*(73)= .34, *p* < .01. While Anglophone students showed very weak positive correlation between family support and wisdom, *r*(151)= .17, *p* < .05. Moreover, for students studying in Anglophone countries, their anxiety was positively related to wisdom, *r*(151)= .24, *p* < .01. However, students in non-Anglophone countries showed no marked correlation, *r*(73)= .08, *p* > .05.

## S6. Current Location: In China or Still Living Abroad During the Pandemic

The rapid of the spread of the virus internationally meant that some students had decided (or their families or home universities asked them) to return home while they could (before borders closed end of March). Therefore, part of our sample had just recently arrived back to China within two weeks when they were asked to respond to the questionnaire. So, we created a binary variable to represent this difference. “Still overseas” (*n* = 145) and “in China” (*n* = 79) were used in the following analyses to tease apart the potential effect of where they were living at that time. We conducted *t*-tests and Pearson’s correlations to examine relationships among the COVID-19 stress, wisdom, coping strategies and anxiety. The results are shown in [Table S9](#TableS7), [Table S10](#TableS8) and [Table S11.](#TableS9)

First, according to [Table S11.](#TableS9), there were stark differences in COVID-19 stress and family support between students who returned to China and those were still in their host countries. Specifically, students in China experienced more COVID-19 stress (*M* = 3.13, *SD* = .75, *p* = .00) than those who were not (*M* = 2.51, *SD* = .76, *p* = .00). Interestingly, this difference is in the opposite of what is expected *t*(223) = -5.76, *p* < .001, *d* = -.77. On top of that, students in China (*t*[223] = -2.45, *p* = .02, *d* = -.33; *M* = 3.71, *SD* = .75, *p* = .02) reported more family support than those who were still living overseas (*M* = 3.43, *SD* = .84, *p* = .02).

Second, while comparing [**Table S9**](#TableS7) and [**Table S10**](#TableS8)**,** students in overseas countries showed a more positive association between COVID-19 school-related stress and anxiety, *r*(79)= .45, *p* < .01, compared to those who had returned to China, *r*(145)= .36, *p* < .01. Furthermore, students overseas experienced a relatively weaker positive relationship between wisdom and ARS coping when compared to students in China, r(79)= .37, *p* < .001, compared to those in China, r(145)= .47, *p* < .001. On top of that, there was moderate positive association between family support and wisdom among students overseas, r(145)= .47, *p* < .01. This is another important finding because Chinese students were potentially seeking out an understanding of what was going on from family back in China as a way to know how to prepare for and combat the pandemic. However, this relationship is not significant among students who returned to China (perhaps due to that stress of gaining and assessing knowledge being relieved by being “back home” and being assured on-site by family or friends).

## S7. Coping Factor Loading:

In order to test the robustness of the Collectivistic Coping Scale (ARS coping and family support), confirmatory factor analysis was conducted, and results are shown in Table S10. Based on the statistics, ARS item 1 (“As a starting point, tried to accept the coronavirus for what it offered me”) to ARS item 11 (“Waited for time to run its course”) loaded in Factor 1 (ARS coping), which means the ARS subscale was valid. However, Family Support subscale seemed less valid. Item 12 (“Shared my feelings with my family”) was load in Factor 1 instead of Factor 2 (Family Support), which meant this item was not stable enough to interpret students’ coping behaviors. Besides, item 13 (“Knew that I could ask assistance from my family increased my confidence”) to item 17 (“Placed trust in my elders’ traditional wisdom to cope with the COVID-19 outbreak”) were cross-loaded in both factors. They can be grouped into Factor 2 (family support) through face validity. It’s possible that this might explain why coping was not associated with the outcome factors in the main text. Researchers should caution this finding.

## S8. Normality test for the main variables

Despite the success test of Q-Q plot and skewness and kurtosis measurement, we also run the Kolmogorov-Smirnov (KS) and Shapiro-Wilk test to further interpret the normal distribution of the variables (presented in Table S14). Unfortunately, both the test yielded significant results and indicated that the residuals of the data did not follow a normal distribution. But according to the central limit theorem, the estimate will have originated from a normal distribution regardless of how the population data are distributed as long as the sample is large enough (approximately greater than 30) (Field, 2018). As such, we are still in confidence of the robustness of the final result.

References

English, A. S. (2020). Facemasks and lockdowns: A move to a new global norm. Paper presented at COVID-19 Social Bridges Conference; July, 21, 2020; Munchen, Germany <https://osf.io/w84z3>

English A.S, Geeraert N. (2020). Crossing the rice-wheat border: Not all intra-cultural adaptation is equal. PLoS ONE15(8): <https://doi.org/10.1371/journal.pone.0236326>

Field, A. (2017). Discovering statistics using IBM SPSS statistics (5th edition). SAGE Publications Ltd.

Goleman, D. (1995). *Emotional intelligence*. New York, NY: Bantam Books.

Plutchik, R. (1982). A psychoevolutionary theory of emotions. *Social Science Information, 21*, 529–553.

Talhelm T, Zhang X, Oishi S, Shimin C, Duan D, Lan X (2014). Large-scale psychological differences within China explained by rice versus wheat agriculture. *Science*. 344(6184):603–8. https://doi.org/10.1126/science.1246850 PMID: 24812395

Tesch, R. (1990). *Qualitative research: Analysis types and software tools*. New York, NY: Falmer Press.

Wei, X. D., & Wang, F. Y. (2020). Southerners are wiser than northerners regarding interpersonal conflicts in China. *Frontiers in Psychology, 11*, 1–8.

Wolcott, H. F. (1994). *Transforming qualitative data: Description, analysis, and interpretation*. Thousand Oaks, CA: Sage.

**Figure S1. The number of participants in various countries around the world during the Coronavirus. Red represents Europe, Blue represents Asia Pacific and Orange represents North America.**


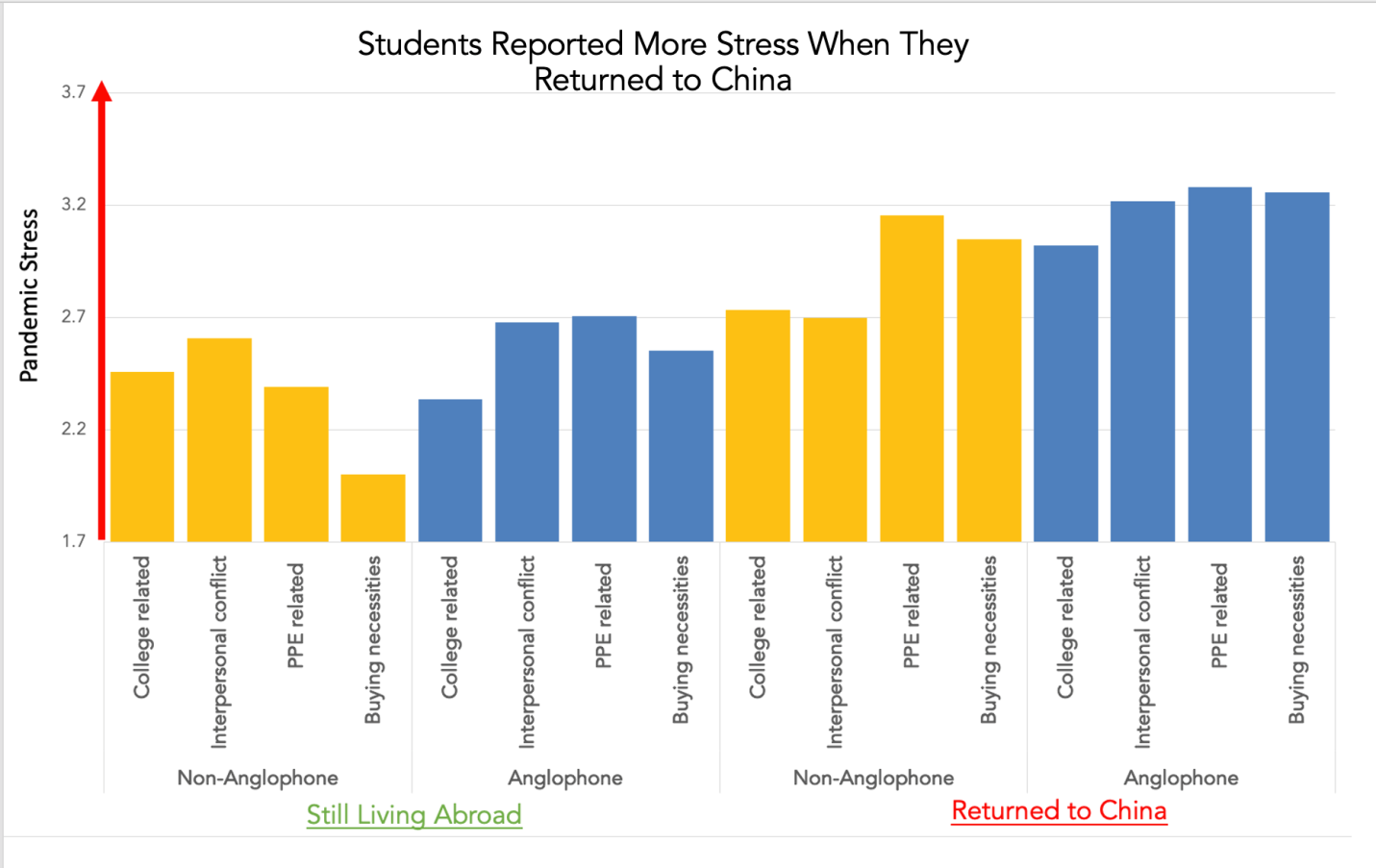


Figure S2. Students reported four different types of social issues, 1) college related; 2) Interpersonal conflict; 3) Personal protective equipment 4) Buying daily necessities. The results show stark differences between students


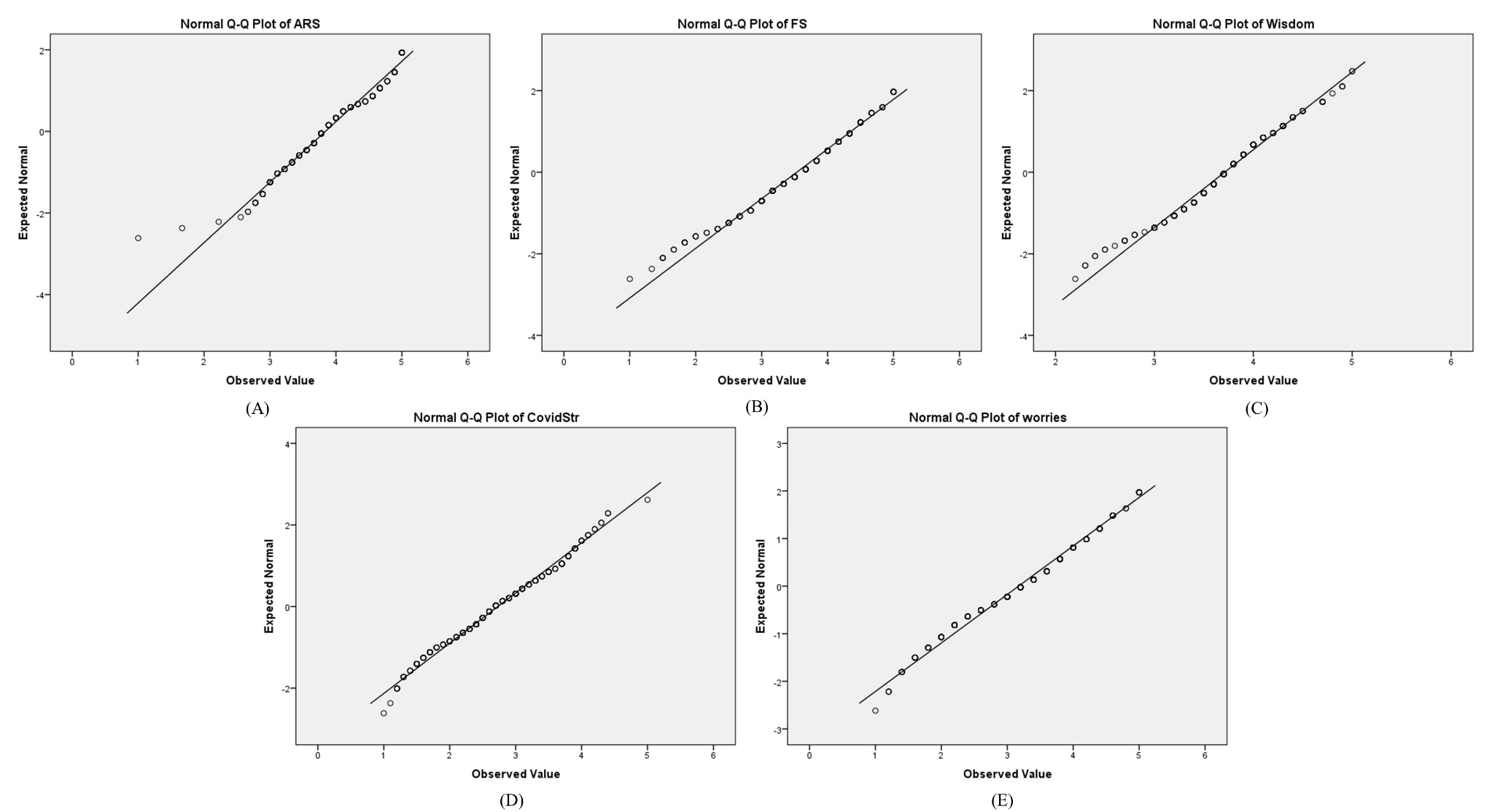


Figure S3. Q-Q Plot of (A) Acceptance, Reframing and Striving; (B) Family Support; (C) Wisdom; (D) COVID-19 Stress; (E) COVID-19 Anxiety

| **Table S1.** The moderating effect of “Acceptance, Reframing and Striving” on the relationship between COVID-19 stress and anxiety | | | | | | |
| --- | --- | --- | --- | --- | --- | --- |
|  | step 1 | | step 2 | | step 3 | |
|  | beta | SE | beta | SE | beta | SE |
| Gender | -0.08 | 0.14 | -0.09 | 0.13 | -0.09 | 0.13 |
| Age | 0.01 | 0.03 | 0.02 | 0.02 | 0.03 | 0.02 |
| Time in host | 0.08 | 0.001 | 0.09 | 0.001 | 0.09 | 0.001 |
| COVID-19 Stress |  |  | 0.41^***^ | 0.06 | 0.41^***^ | 0.06 |
| ARS Coping |  |  | -0.07 | 0.06 | -0.08 | 0.06 |
| ARS Coping × COVID-19 |  |  |  |  | -0.05 | 0.06 |
| **model statistics** |  |  |  |  |  |  |
| *R^2^* | 0.01 | | 0.19 | | 0.19 | |
| *ΔR^2^* | 0.01 | | 0.18 | | 0.002 | |
| *F* | 0.85 | | 24.27 | | 0.64 | |
| *df* | (3, 220) | | (2, 218) | | (1, 217) | |
| Note. ^***^ *p*<.001; Gender is coded (1=Male, 0=Female). ARS, Acceptance, Reframing and Striving. ARS Coping and COVID-19 Stress were standardized in prior. | | | | | | |

| **Table S2.** The moderating effect of family support on the relationship between COVID-19 stress and anxiety | | | | | | |
| --- | --- | --- | --- | --- | --- | --- |
|  | step 1 | | step 2 | | step 3 | |
|  | beta | SE | beta | SE | beta | SE |
| Gender | -0.08 | 0.14 | -0.09 | 0.13 | -0.09 | 0.13 |
| Age | 0.01 | 0.03 | 0.02 | 0.02 | 0.03 | 0.02 |
| Time in host | 0.08 | 0.001 | 0.09 | 0.001 | 0.08 | 0.001 |
| COVID-19 Stress |  |  | 0.42^***^ | 0.06 | 0.45^***^ | 0.06 |
| Family Support |  |  | -0.03 | 0.06 | -0.03 | 0.06 |
| Family Support × COVID-19 |  |  |  |  | -0.09 | 0.06 |
| **model statistics** |  |  |  |  |  |  |
| *R^2^* | 0.01 | | 0.19 | | 0.20 | |
| *ΔR^2^* | 0.01 | | 0.18 | | 0.01 | |
| *F* | 0.85 | | 23.67 | | 2.08 | |
| *df* | (3, 220) | | (2, 218) | | (1, 217) | |
| Note. ^***^*p*<.001; Gender is coded (1=Male, 0=Female). Family Support and COVID-19 Stress were standardized in prior. | | | | | | |

**Table S3**. Demographic variables

|  | Non-Anglophone  (n=73) | |  | Anglophone  (n=151) | |  | Total | |
| --- | --- | --- | --- | --- | --- | --- | --- | --- |
|  | *M* | *SD* |  | M | *SD* |  | *M* | *SD* |
| Age | 24.33 | 3.13 |  | 23.68 | 2.35 |  | 23.89 | 2.64 |
| Gender | N | % |  | N | % |  | N | % |
| *Male* | 23 | 31.5% |  | 59 | 39.1% |  | 82 | 36.6% |
| *Female* | 50 | 68.5% |  | 92 | 60.9% |  | 142 | 63.4% |
| Residence length |  |  |  |  |  |  |  |  |
| *Year* | 1.38 | 1.63 |  | 1.66 | 2.70 |  | 1.57 | 2.40 |
| *Month* | 5.00 | 2.99 |  | 6.00 | 2.70 |  | 5.67 | 2.83 |
| Education level |  |  |  |  |  |  |  |  |
|  | N | % |  | N | % |  | N | % |
| *Undergraduate* | 18 | 24.7% |  | 37 | 24.5% |  | 55 | 24.6% |
| *Postgraduate* | 41 | 56.2% |  | 100 | 66.2% |  | 141 | 62.9% |
| *PhD* | 11 | 15.1% |  | 14 | 9.3% |  | 25 | 11.2% |
| *Others* | 3 | 4.1% |  | 0 | 0 |  | 3 | 1.3% |
| Response date | N | % |  | N | % |  | N | % |
| *2020/04/03* | 35 | 47.9% |  | 77 | 51.0% |  | 112 | 50.0% |
| *2020/04/04* | 9 | 12.3% |  | 18 | 11.9% |  | 27 | 12.1% |
| *2020/04/05* | 19 | 26.0% |  | 22 | 14.6% |  | 41 | 18.3% |
| *2020/04/06* | 9 | 12.3% |  | 27 | 17.9% |  | 36 | 16.1% |
| *2020/04/07* | 1 | 1.4% |  | 4 | 2.6% |  | 5 | 2.2% |
| *2020/04/08* | 0 | 0 |  | 3 | 2.0% |  | 3 | 1.3% |

| **Table S4. Factor Loadings of COVID-19 Stress Scale** | | |
| --- | --- | --- |
| How stressed do you feel about …. | COVID-19 Stress Dimensions | |
|  | School Stress | Daily life stress |
| 1. Have to wear masks |  | .63 |
| 1. Fear of discrimination for wearing a mask |  | .67 |
| 1. Feel unsafe in this city |  | .60 |
| 1. Go out and buy daily necessities |  | .65 |
| 1. Read the news on the spreading of the outbreak |  | .56 |
| 1. Have to stay indoors | .56 | .42 |
| 1. School shutdown | .72 |  |
| 1. Finish academic assignments or encounter graduation issues | .72 |  |
| 1. Unable to return to China | .52 |  |
| 1. Have problems in finding/staying in my accommodation | .42 |  |
| Note: Principal Component Analysis was conducted with Varimax rotation.  Loadings below .40 were removed | | |

**Table S5**. Mean analysis for students back to China and not in China

|  | Not in China (*n* =145) | |  | In China (*n* =79) | |  | Total | |
| --- | --- | --- | --- | --- | --- | --- | --- | --- |
|  | *M* | *SD* |  | *M* | *SD* |  | *M* | *SD* |
| Covid-19 stressors | 2.51 | .76 |  | 3.13 | .75 |  | 2.73 | .81 |
| (Subscale) Daily stress | 2.61 | .89 |  | 3.13 | .82 |  | 2.79 | .90 |
| (Subscale) School stress | 2.37 | .82 |  | 3.13 | .88 |  | 2.64 | .92 |
| Degree of trauma | 1.34 | .76 |  | 1.21 | .58 |  | 1.29 | .70 |
| Degree of emotion | 1.13 | .48 |  | 1.11 | .42 |  | 1.12 | .46 |
| General emotion | 3.09 | 1.38 |  | 2.65 | 1.13 |  | 2.94 | 1.31 |
|  | *N* | % |  | *N* | % |  | *N* | % |
| Joy | 1 | .7% |  | 1 | 1.3% |  | 2 | .9% |
| Fear | 80 | 55.2% |  | 54 | 68.4% |  | 134 | 59.8% |
| Sadness | 11 | 7.6% |  | 5 | 6.3% |  | 16 | 7.1% |
| Anger | 5 | 3.4% |  | 7 | 8.9% |  | 12 | 5.4% |
| Neutral | 45 | 31.0% |  | 11 | 13.9% |  | 56 | 25.0% |
| General event | 2.46 | 1.23 |  | 2.84 | 1.12 |  | 2.60 | 1.20 |
|  | *N* | *%* |  | *N* | *%* |  | *N* | % |
| School related event | 42 | 29.0% |  | 12 | 15.2% |  | 54 | 24.1% |
| Interpersonal relationship | 27 | 18.6% |  | 16 | 20.3% |  | 43 | 19.2% |
| PPE-related event | 37 | 25.5% |  | 22 | 27.8% |  | 59 | 26.3% |
| Daily stuff buying | 25 | 17.2% |  | 24 | 30.4% |  | 49 | 21.9% |

**Table S6.** Correlations of Key variables Studying in “Non-Anglophone”

|  | | | | Correlations | | | | | | | | | | | | | | | | | | | |  | |  | |  |
| --- | --- | --- | --- | --- | --- | --- | --- | --- | --- | --- | --- | --- | --- | --- | --- | --- | --- | --- | --- | --- | --- | --- | --- | --- | --- | --- | --- | --- |
| Study Location | | | | 1 | | 2 | | 3 | | 4 | | 5 | | 6 | | 7 | | 8 | | 9 | | 10 | | 11 | | 12 | |  |
| Non-Anglophone Country  (n = 73) | 1. | COVID-19 Stress | - | |  | |  | |  | |  | |  | |  | |  | |  | |  | |  | |  | |  | |
|  | 2. | COVID Daily | .92^***^ | | - | |  | |  | |  | |  | |  | |  | |  | |  | |  | |  | |  | |
|  | 3. | COVID School | .81^***^ | | .50^***^ | | - | |  | |  | |  | |  | |  | |  | |  | |  | |  | |  | |
|  | 4. | Wisdom | .11 | | .05 | | .17 | | - | |  | |  | |  | |  | |  | |  | |  | |  | |  | |
|  | 5. | ARS | -.09 | | -.14 | | .00 | | .36^**^ | | - | |  | |  | |  | |  | |  | |  | |  | |  | |
|  | 6. | Family Support | .19 | | .16 | | .17 | | .34^**^ | | .41^***^ | | - | |  | |  | |  | |  | |  | |  | |  | |
|  | 7. | Total Coping | .04 | | .00 | | .09 | | .41^***^ | | .87^***^ | | .78^***^ | | - | |  | |  | |  | |  | |  | |  | |
|  | 8. | Anxiety | .43^***^ | | .44^***^ | | .29^*^ | | .08 | | -.12 | | -.01 | | -.09 | | - | |  | |  | |  | |  | |  | |
|  | 9. | Degree of Trauma | -.04 | | -.03 | | -.05 | | .06 | | .15 | | .11 | | .16 | | .13 | | - | |  | |  | |  | |  | |
|  | 10. | Degree of Emotion | -.01 | | .02 | | -.04 | | .06 | | -.05 | | -.10 | | -.08 | | .30^*^ | | .16 | | - | |  | |  | |  | |
|  | 11. | General Emotion | -.22 | | -.20 | | -.19 | | -.01 | | .07 | | -.18 | | -.03 | | -.07 | | -.26^*^ | | .11 | | - | |  | |  | |
|  | 12. | General Event | .06 | | -.03 | | .17 | | .00 | | .06 | | .21 | | .12 | | -.13 | | -.13 | | -.21 | | -.58^***^ | | - | |  | |

Note: *p<0.05 (2-tailed); **p<0.01 (2-tailed); ***p<0.001 (2-tailed)

ARS Coping “acceptance, reframing and striving”

| **Table S7.** Correlations of Key variables Studying in “Anglophone” | | | | | | | | | | | | | | |  |
| --- | --- | --- | --- | --- | --- | --- | --- | --- | --- | --- | --- | --- | --- | --- | --- |
|  |  |  | *Correlations* | | | | | | | | | | | |  |
| Study Location | | | 1 | 2 | 3 | 4 | 5 | 6 | 7 | 8 | 9 | 10 | 11 | 12 | |
| Anglophone Country  (n = 151) | 1 | COVID-19 Stress | - |  |  |  |  |  |  |  |  |  |  |  | |
|  | 2 | COVID Daily | .94^***^ | - |  |  |  |  |  |  |  |  |  |  | |
|  | 3 | COVID School | .86^***^ | .62^***^ | - |  |  |  |  |  |  |  |  |  | |
|  | 4 | Wisdom | -.03 | -.06 | .01 | - |  |  |  |  |  |  |  |  | |
|  | 5 | ARS | -.14 | -.16 | -.07 | .43^***^ | - |  |  |  |  |  |  |  | |
|  | 6 | Family Support | .02 | -.04 | .11 | .17^*^ | .30^***^ | - |  |  |  |  |  |  | |
|  | 7 | Total Coping | -.08 | -.13 | .01 | .40^***^ | .86^***^ | .72^***^ | - |  |  |  |  |  | |
|  | 8 | Anxiety | .50^***^ | .44^***^ | .48^***^ | .24^**^ | -.01 | -.05 | .00 | - |  |  |  |  | |
|  | 9 | Degree of Trauma | .16^*^ | .23^**^ | .03 | .09 | .08 | .08 | .08 | .10 | - |  |  |  | |
|  | 10 | Degree of Emotion | .05 | .09 | -.04 | .02 | -.03 | .01 | -.02 | .10 | .28^**^ | - |  |  | |
|  | 11 | General Emotion | -.26^**^ | -.22^**^ | -.27^**^ | -.13 | -.08 | -.07 | -.10 | -.18^*^ | -.08 | -.11 | - |  | |
|  | 12 | General Event | .12 | .10 | .13 | .09 | .00 | .07 | .05 | -.03 | -.07 | .09 | -.47^***^ | - | |
| Note: *p<0.05 (2-tailed); **p<0.01 (2-tailed); ***p<0.001 (2-tailed)  ARS Coping “acceptance, reframing and striving” | | | | | | | | | | | | | | |  |

**Table S8.** Results of independent *t*-test between Anglophone and non-Anglophone

| Variables | *t* | *p* | Non-Anglophone  (n=73) | |  | Anglophone  (n=151) | |
| --- | --- | --- | --- | --- | --- | --- | --- |
|  |  |  | *M* | *SD* |  | *M* | *SD* |
| Covid-19 stressors | -.81 | .42 | 2.67 | .85 |  | 2.76 | .80 |
| *Daily stressors* | -.63 | .53 | 2.73 | .96 |  | 2.82 | .87 |
| *School-related stressors* | -.85 | .39 | 2.57 | .99 |  | 2.68 | .89 |
| Wisdom | 1.07 | .29 | 3.76 | .50 |  | 3.68 | .54 |
| *Acceptance, reframing, and striving* | 1.29 | .20 | 3.92 | .72 |  | 3.80 | .65 |
| *Family support* | 1.24 | .22 | 3.63 | .91 |  | 3.49 | .78 |
| Anxiety | -2.48* | .01 | 2.94 | 1.08 |  | 3.28 | .91 |
| Degree of trauma | -1.46 | .15 | 1.19 | .64 |  | 1.34 | .73 |
| Degree of emotion | 1.84 | .07 | 1.21 | .58 |  | 1.09 | .38 |

Notes: *M*, means; *SD*, standard deviation; *p* < .05 means statistically significant.

**Table S9.** Correlations of Key variables in China

|  | | | *Correlations* | | | | | | | | | | | |
| --- | --- | --- | --- | --- | --- | --- | --- | --- | --- | --- | --- | --- | --- | --- |
| Current location | | | 1 | 2 | 3 | 4 | 5 | 6 | 7 | 8 | 9 | 10 | 11 | 12 |
| In China  (n = 79) | 1. | COVID-19 Stress | - |  |  |  |  |  |  |  |  |  |  |  |
|  | 2. | COVID Daily | .93^***^ | - |  |  |  |  |  |  |  |  |  |  |
|  | 3. | COVID School | .80^***^ | .52^***^ | - |  |  |  |  |  |  |  |  |  |
|  | 4. | Wisdom | -.04 | -.08 | .03 | - |  |  |  |  |  |  |  |  |
|  | 5. | ARS | -.15 | -.16 | -.08 | .37^***^ | - |  |  |  |  |  |  |  |
|  | 6. | Family Support | -.05 | -.07 | .01 | .16 | .31^***^ | - |  |  |  |  |  |  |
|  | 7. | Total Coping | -.12 | -.14 | -.04 | .35^***^ | .86^***^ | .72^***^ | - |  |  |  |  |  |
|  | 8. | Anxiety | .48^***^ | .40^***^ | .45^**^ | .17^*^ | -.06 | -.05 | -.05 | - |  |  |  |  |
|  | 9. | Degree of Trauma | .11 | .15 | .01 | .08 | .11 | .09 | .11 | .13 | - |  |  |  |
|  | 10. | Degree of Emotion | .07 | .09 | .02 | .07 | -.07 | .02 | -.03 | .20^*^ | .24^**^ | - |  |  |
|  | 11. | General Emotion | -.20^*^ | -.20^*^ | -.14 | -.12 | .07 | -.03 | .01 | -.14 | -.14 | -.06 | - |  |
|  | 12. | General Event | .03 | .04 | .01 | .07 | .00 | .07 | .03 | -.09 | -.03 | -.01 | -.49^***^ | - |

Note: *p<0.05 (2-tailed); **p<0.01 (2-tailed); ***p<0.001 (2-tailed)

| **Table S10.** Correlations of Key variables Recently Overseas | | | | | | | | | | | | | | |  |
| --- | --- | --- | --- | --- | --- | --- | --- | --- | --- | --- | --- | --- | --- | --- | --- |
|  |  | Correlations | | | | | | | | | | | | |  |
| Current location | | | 1 | 2 | 3 | 4 | 5 | 6 | 7 | 8 | 9 | 10 | 11 | 12 | |
| Overseas  (n= 145) | 1. | COVID-19 Stress | - |  |  |  |  |  |  |  |  |  |  |  | |
|  | 2. | COVID Daily | .92^***^ | - |  |  |  |  |  |  |  |  |  |  | |
|  | 3. | COVID School | .84^***^ | .56^***^ | - |  |  |  |  |  |  |  |  |  | |
|  | 4. | Wisdom | .01 | -.01 | .01 | - |  |  |  |  |  |  |  |  | |
|  | 5. | ARS | -.22 | -.25^*^ | -.12 | .47^***^ | - |  |  |  |  |  |  |  | |
|  | 6. | Family Support | .17 | .12 | .20 | .35^**^ | .40^***^ | - |  |  |  |  |  |  | |
|  | 7. | Total Coping | -.06 | -.10 | .01 | .51^***^ | .87^***^ | .78^***^ | - |  |  |  |  |  | |
|  | 8. | Anxiety | .51^***^ | .51^***^ | .36^**^ | .15 | -.09 | -.08 | -.10 | - |  |  |  |  | |
|  | 9. | Degree of Trauma | .24^*^ | .27^*^ | .12 | .08 | .08 | .11 | .11 | .13 | - |  |  |  | |
|  | 10. | Degree of Emotion | -.07 | .00 | -.15 | -.01 | .08 | -.12 | -.03 | .07 | .11 | - |  |  | |
|  | 11. | General Emotion | -.27^*^ | -.11 | -.33^**^ | .02 | -.22 | -.19 | -.21 | -.13 | -.20 | .10 | - |  | |
|  | 12. | General Event | .09 | -.03 | .24^*^ | .00 | .02 | .15 | .09 | -.07 | -.17 | -.07 | -.50^***^ | - | |
| Note: *p<0.05 (2-tailed); **p<0.01 (2-tailed); ***p<0.001 (2-tailed) | | | | | | | | | | | | | | |  |

**Table S11.** Results of independent *t*-test between students back in China and not in China

| Variables | *t* | *p* | Not in China  (*n*=145) | |  | In China  (*n*=79) | |
| --- | --- | --- | --- | --- | --- | --- | --- |
|  |  |  | *M* | *SD* |  | *M* | *SD* |
| Covid-19 stressors | -5.76 | .00 | 2.51 | .76 |  | 3.13 | .75 |
| Daily stressors | -4.27 | .00 | 2.61 | .89 |  | 3.13 | .82 |
| School-related stressors | -6.33 | .00 | 2.38 | .83 |  | 3.13 | .88 |
| Wisdom | -1.40 | .16 | 3.67 | .54 |  | 3.78 | .49 |
| Acceptance, reframing, and striving | -1.40 | .16 | 3.79 | .71 |  | 3.92 | .60 |
| Family support | -2.45 | .02 | 3.43 | .84 |  | 3.71 | .75 |
| Anxiety | -.45 | .66 | 3.15 | 1.01 |  | 3.21 | .93 |
| Degree of trauma | 1.38 | .17 | 1.34 | .76 |  | 1.20 | .59 |
| Degree of emotion | .27 | .79 | 1.13 | .48 |  | 1.11 | .42 |

| **Table S12. Factor Loadings of the Collectivistic Coping Scale** | | |
| --- | --- | --- |
| To what degree did you use the following ways to cope with COVID-19 stress in the past two weeks? | Collectivistic Coping Dimensions | |
|  | ARS coping | Family support |
| 1. As a starting point, tried to accept the coronavirus for what it offered me | .54 |  |
| 1. Believed that I would grow from surviving the outbreak of covid-19 | .65 |  |
| 1. Told myself that I could make my plans and ideas work | .64 |  |
| 1. Told myself that I could think of effective ideas | .71 |  |
| 1. Realized that the covid-19 pandemic served as an important purpose in my life | .58 |  |
| 1. Analyzing my feelings provided me with ideas about how to proceed | .66 |  |
| 1. Realized that often good comes after overcoming bad situations | .61 |  |
| 1. Maintained good relationships with people around me | .61 |  |
| 1. Not vented my negative feelings to some people around me | .43 |  |
| 1. Accepted the pandemic outbreak as fate | .40 |  |
| 1. Waited for time to run its course | .49 |  |
| 1. Shared my feelings with my family | .55 |  |
| 1. Knew that I could ask assistance from my family increased my confidence | .62 | .51 |
| 1. Through family assistance and support confidence | .51 | .65 |
| 1. Followed the norms and expectations of my family about handling the covid-19 outbreak | .60 | .47 |
| 1. Followed the guidance of my elders (e.g., parents and older relatives) | .53 | .61 |
| 1. Placed trust in my elders’ traditional wisdom to cope with the COVID-19 outbreak | .50 | .60 |
| Note: Principal Component Analysis was conducted with Varimax rotation. Loadings below .40 were removed | | |

| **Table S13.** People from Rice areas report *less* wise-reasoning | | | | |
| --- | --- | --- | --- | --- |
|  | Demographic Model | | Rice Model | |
|  | *b* | P | *b* | p |
| Current Location | .09 | .17 | .10 | .15 |
| Study Location | -.06 | .37 | -.04 | .53 |
| Educational Level | .04 | .60 | .04 | .53 |
| Time in Host Country | .01 | .84 | .01 | .94 |
| Prefecture Rice Farming |  |  | -.14 | .04 |
| **model statistics** |  | |  | |
| *F (df)* | .79*(219)* | | 1.50*(218)* | |
| *R^2^* | .01 | | .03 | |
| *ΔR^2^* | .00 | | .01 | |
| Note. Dependent variable: Wisdom; Current Location (In China =1, Not in China =0); Study Location (Studying in an Anglophone Country =1, Studying in a non-Anglophone Country = 0); Education level (Bachelors =1, Masters Degree =2, PhD = 4, 5 = Other like exchange or Language student) | | | | |

| **Table S14.** Tests of Normality | | | | |
| --- | --- | --- | --- | --- |
|  | Kolmogorov-Smirnov | | Shapiro-Wilk | |
|  | Statistic | P | Statistic | P |
| ARS | .08 | .004 | .97 | .000 |
| Family support | .08 | .003 | .98 | .001 |
| COVID-19 anxiety | .09 | .000 | .98 | .001 |
| Wise-reasoning | .09 | .000 | .98 | .005 |
| COVID-19 Stress | 0.6 | .01 | .99 | .040 |
| Note. Dependent variable: ARS, Acceptance, Reframing, and Striving; | | | | |
